# Recently Identified Biomarkers That Promote Lymph Node Metastasis in Head and Neck Squamous Cell Carcinoma

**DOI:** 10.3390/cancers3010747

**Published:** 2011-02-22

**Authors:** Elyse L. Walk, Scott A. Weed

**Affiliations:** Department of Neurobiology and Anatomy, Program in Cancer Cell Biology, Mary Babb Randolph Cancer Center, West Virginia University, Morgantown, WV 26506, USA; E-Mail: ewalk@hsc.wvu.edu

**Keywords:** head and neck cancer, lymph node, invasion, metastasis, biomarker

## Abstract

Head and neck squamous cell carcinoma (HNSCC) is a heterogeneous cancer that arises in the upper aerodigestive tract. Despite advances in knowledge and treatment of this disease, the five-year survival rate after diagnosis of advanced (stage 3 and 4) HNSCC remains approximately 50%. One reason for the large degree of mortality associated with late stage HNSCC is the intrinsic ability of tumor cells to undergo locoregional invasion. Lymph nodes in the cervical region are the primary sites of metastasis for HNSCC, occurring before the formation of distant metastases. The presence of lymph node metastases is strongly associated with poor patient outcome, resulting in increased consideration being given to the development and implementation of anti-invasive strategies. In this review, we focus on select proteins that have been recently identified as promoters of lymph node metastasis in HNSCC. The discussed proteins are involved in a wide range of critical cellular functions, and offer a more comprehensive understanding of the factors involved in HNSCC metastasis while additionally providing increased options for consideration in the design of future therapeutic intervention strategies.

## 1. Introduction

The main cause of cancer-related death is due to metastasis of primary tumors to secondary sites within the body. Advanced cases of head and neck squamous carcinoma (HNSCC) primarily spread locoregionally, where tumor cells infiltrate the lymphatic drainage and travel into cervical lymph nodes [1]. While incidence rates and overall disease-related deaths have dropped over recent years [2], the five-year survival rate for patients presenting with clinically advanced disease remains around 55% [3]. Predicting the inherent metastatic potential of primary HNSCC tumors would serve to aid in enhancing approaches to treatment that would improve patient management. However, current diagnosing strategies rely in part on histological analyses of biopsy samples, which have largely proven inadequate due to the high frequency of patients with recurrent disease [4]. The presence of lymph node metastasis in HNSCC patients has long been established as a poor prognostic indicator [5-7], making earlier detection of tumors with the propensity to invade and spread through local lymphatics an important step in patient management towards a more promising outcome. The stepwise model of carcinoma progression involves changes at the molecular level that ultimately provide normal epithelia cells with the ability to invade surrounding tissue [8]. In HNSCC, efforts at identifying molecules associated with and responsible for driving nodal metastasis has revealed many potential biomarkers for this process over the past decade [4,9-11]. Some of the more notable markers associated with nodal metastasis are cell cycle and proliferation regulators such as p53, epidermal growth factor receptor (EGFR), p16, and cyclin D1. The roles of these proteins in HNSCC development and progression are well documented [4,11-13,13]. More recently, expression profiling through DNA microarray technology has been useful in identifying genes previously unrecognized in the field that also contribute to or are associated with lymph node metastasis [14-16]. In this review, we focus on various studies conducted within the past four years that have linked overexpression of specific proteins in HNSCC to lymph node metastasis, highlighting several new potential candidates (Table 1) that could prove useful in the prediction, detection and treatment determination of metastatic disease.

## 2. Proteins Involved in Cell Cycle Regulation, Proliferation and Apoptosis

Regulation of the cell cycle requires the coordination of many protein classes, creating a system of checks and balances that when dysregulated results in either cell proliferation or death. Mutations or altered expression of proteins important for proper governing of cell cycle initiation and coordination can set the system off balance, providing tumor cells with means to bypass normal cell cycle check points, evade apoptosis and over-proliferate. Several proteins involved in cell cycle regulation have recently been identified as markers with increased expression in HNSCC that correspond with lymph node metastasis.

### 2.1. c-Met

The receptor tyrosine kinase c-Met is normally active during embryonic development and wound healing [17]. Activated c-Met promotes cellular proliferation by organizing an “invasive growth” program by which normal cells migrate to new sites to form polarized cells and functional 3D structures. [18]. The ligand for c-Met is hepatocyte growth factor (HGF). HGF is secreted by mesenchymal cells and upon binding to c-Met causes receptor homodimerization and phosphorylation/activation of the catalytic site, resulting in downstream signaling events that lead to cell transformation and invasion [17,19]. Expression of c-Met on epithelial cells enables them to receive signals from surrounding stromal cells through stromal cell HGF secretion. In transformed cells, c-Met activity is enhanced through several mechanisms, including increased ligand-based stimulation via elevated autocrine or paracrine HGF secretion, receptor overexpression or mutational activation of the c-Met kinase domain [17,19]. One notable function of c-Met is its interaction with the oncogenic tyrosine kinase c-Src, creating a mechanism to bypass inhibited EGFR signaling in breast cancer cell lines [20] as well as increasing resistance to c-Src inhibitory drugs in HNSCC [21]. Both c-Src and EGFR are overexpressed in HNSCC and are important in head and neck cancer development and progression [22]. A retrospective analysis of 61 surgically treated cases of HNSCC showed frequent expression of c-Met in tumors with higher T-stage classification [23]. Patients with lymph node metastasis have a significant increase in c-Met expression when compared with indolent cases lacking metastasis. While HGF was found at elevated levels in over 60% of the cases, it was not a significant factor in this study; however when combined with c-Met overexpression there was a correlation with lymph node metastasis. Being a paracrine factor secreted by cells of mesenchymal origin, HGF may be more relevant as a serum biomarker, as shown in previous studies [24-26]. In these prospective studies, serum cytokine levels were measured in patients before and after treatment. High levels of serum HGF were found in patients before treatment, decreased after treatment, and were found to increase again with recurrence. These results suggest monitoring HGF levels may prove useful in determining response to treatment and recurrence. The HGF/c-Met pathway has been implicated in invasion and metastasis in HNSCC and other cancers, both *in vitro* and *in vivo* [27-31] and has been linked to resistance of EGFR inhibitors and cisplatin [20,32-35], making both c-Met and HGF attractive drug targets as well as determinants of treatment. Currently there are several clinical trials involving drugs that target c-Met or HGF specifically, although these trials have not gone past phase I/II [36].

### 2.2. CEP55 (FLJ10540)

The cytokinesis regulator CEP55, also known as FLJ10540, is a 55 kDa protein that localizes to the centrosome of chromosomes in interphase and the midbody during cytokinesis, where it mediates the final stages of mitotic division into two daughter cells [37]. CEP55 is a recently identified downstream target of the oncogene FOXM1, which has been shown to be upregulated in pre-malignant HNSCC lesions [38]. Subsequently, CEP55 overexpression has been directly correlated with an increase in tumor aggressiveness in oral squamous cell carcinoma (OSCC) [39]. Retrospective immunohistochemistry (IHC) analysis revealed overexpression in patient tumor samples, which was linked to tumor and nodal stage as well as a poor prognosis [39]. There was also significantly higher expression in patients with advanced T stage (3 and 4) with lymph node metastasis when compared with node negative and stage 1-2 tumors. *In vitro* work has linked CEP55 expression to increased cell motility and invasion through regulation of FOXM1 and MMP-2 [39]. In another report, while CEP55 was shown to be significantly upregulated in dysplasias and HNSCC, upregulation within lymph node metastases was not significant, which the authors cite as being due to tissue heterogeneity [40]. Taken together, these results suggest CEP55 may prove useful in predicting disease progression. As cytokinesis is of obvious importance to highly proliferative cells, overexpression of CEP55 is therefore a logical candidate for potential use as an HNSCC metastatic biomarker in clinical settings.

### 2.3. NBS1

Nijmegen breakage syndrome (NBS) is a syndrome characterized by growth retardation, immunodeficiencies and predisposition to malignancies [41]. The only gene associated with this syndrome is NBS1, and its gene product plays an important cell cycle checkpoint role in double strand DNA break repair [41]. NBS1 is part of a complex including Mre11 and Rad5 (MRN complex) that is central to detection of DNA breakage, coordinating response programs for and catalyzing repair mechanisms of double-strand breaks [42]. A study analyzing OSCC samples revealed an increase in NBS1 mRNA expression that correlated to increased protein expression [43]. NBS1 overexpression was associated with advanced disease and recurrence/metastasis in OSCC, while non-oral HNSCC samples with the same levels of expression were only associated with recurrence. An increase in NBS1 expression in HNSCC regardless of origin site was additionally associated with lymph node involvement [43]. In this same study, NBS1 was found to be a prognostic marker even with samples divided into subgroups based on tumor and nodal stage or treatment type. Earlier studies by the authors had linked NBS1 overexpression to more aggressive disease and worse prognosis in advanced HNSCC [44], and to lymph node and distant metastasis [45]. These studies also determined NBS1 expression to be involved in cellular transformation through activation of the PI3K/Akt pathway and induction of EMT [44,45]. One explanation for this association could be due to single nucleotide polymorphisms (SNPs) within the NBS1 gene. Several studies have linked genetic variations to development of cancers of the breast, lung, esophagus, non-Hodgkin's lymphoma and upper aerodigestive tract [46-48]. Identifying high risk patients through detection of NBS1 SNPs may be a useful tool in predicting patient outcome in OSCC and other HNSCC subtypes.

### 2.4. Survivin

An inhibitor of apoptosis (IAP) family member, survivin, suppresses apoptosis by directly binding to and inhibiting caspase family members, typically caspase 3 and caspase 7, or by indirectly suppressing apoptosis through activation of caspase-associated cofactors [49,50]. Survivin overexpression has been identified in different cancer types, suggesting it may be a tumor marker and possible drug target [49,50]. There have been multiple studies linking survivin expression to HNSCC progression in recent years. In a retrospective study of 42 OSCC cases, individuals with lymph node metastasis had significantly high survivin expression compared to non-metastatic cases [51]. A correlation between expression and low survival rate was also concluded in this study. A significantly higher expression of survivin was found in another study of OSCC cases [52]. Premalignant lesions were also included, and survivin expression was elevated in these regions when compared to normal tissue. Survivin has been found to play different roles depending on location within the cell [49]. In the nucleus, survivin controls cell division by functioning as a subunit of the chromosomal passenger complex (CPC), while cytoplasmic survivin is cytoprotective, providing the cell with protection from cell death induced by radiation or chemotherapeutic drugs [53]. An examination of normal and HNSCC tissues by IHC showed nuclear and cytoplasmic staining, both which were significantly correlated to poor differentiation and lymph node metastasis [54]. There have been several other studies that investigated the prognostic role of cytoplasmic and nuclear survivin. Although no general consensus was found based on subcellular location, survivin expression has consistently been linked to unfavorable outcome and reduced disease-free survival. [55-57]. The anti-apoptotic effects of survivin may be linked to treatment failure as some *in vitro* studies have suggested [58-60]. In one particular model, HNSCC cells that escaped cellular senescence after treatment with the chemotherapeutic drug camptothecin were unable to escape senescence upon knockdown of survivin [61]. Another retrospective study analyzed OSCC cases and correlated survivin mRNA expression to tumor differentiation, stage and lymph node involvement. This study also found that down-regulation of survivin increased sensitivity of HNSCC cells to cisplatin [62]. While more studies are needed to firmly link survivin overexpression to lymph node metastasis, its role as a prognostic and drug resistance marker warrants further investigation as a potential therapeutic target.

### 2.5. RSK2

p90 ribosomal S6 kinase 2 (RSK2) is a serine/threonine kinase activated downstream in the MAPK pathway [63,64]. Numerous substrates have been identified for RSK2, including GSK3β, c-Fos, p27^kip1^, e1F4B, and p65, a subunit of NF-κB. These substrates link RSK2 activity in mediating pathways central to cell proliferation, transcriptional and translational regulation, survival and apoptosis [63,64]. Retrospective IHC analysis has identified RSK2 overexpression in HNSCC tumor and lymph node patient samples [65]. Primary tumors from patients with lymph node metastases and matched lymph node specimens had a significantly higher expression of RSK2 than patients with non-metastatic primary HNSCC. Manipulation of RSK2 levels by RNA interference demonstrates a clear dependence of RSK2 expression levels in modulating invasion in Matrigel transwell assays. Similar results were obtained in a xenograft mouse model, where cells with stable knockdown of RSK2 had less metastatic potential than controls [65]. Previous *in vitro* work identified RSK2 as a critical regulator in cellular transformation [66] and though not extensively studied in head and neck cancer, the versatility of RSK2 makes it a worthwhile target for additional investigation. Multiple small molecule inhibitors specific to RSK family members have been identified [67] that may serve as viable platforms for further development of potential targeted anti-RSK2 therapeutic compounds.

## 3. Cell Motility, Adhesion and Extracellular Matrix Degradation

Without the ability to modify their surroundings and move to new areas, tumor cells would remain in their primary location, making them more manageable by conventional surgical and radiation-based treatment regimes. Overexpression of proteins involved in cell motility and extracellular matrix remodeling equips primary HNSCC cells with the ability to degrade and escape an encapsulating extracellular matrix and penetrate through the surrounding stroma, ultimately resulting in lymphatic intravasation and spread into regional lymph nodes.

### 3.1. Cortactin

Cortactin is an actin binding protein that plays roles in cell motility and invasion by promoting Arp2/3 complex actin nucleation and by stabilizing the newly formed actin branchpoints [68]. Located at chromosomal region 11q13, an area frequently amplified in HNSCC, cortactin has consistently been associated with more aggressive and invasive tumors, lymph node metastasis and poor clinical outcome in HNSCC [69-73]. Yamada *et al.* analyzed a series of OSCC patient biopsy samples, finding overexpression of cortactin more often in OSCC than in normal epithelium, as well as localization of cortactin at the invasive front [74]. Cortactin overexpression was also found more frequently in tumors with high T and N classification and significantly correlated to regional invasion in these patients [74]. A separate study looked at the relationship between CTTN gene amplification status, mRNA and protein expression in patients with pharyngeal or laryngeal SCC [75]. This study found that gene amplification correlated significantly to mRNA and protein expression, with cases containing strong cortactin staining significantly associated with lymph node metastasis. This group conducted an analysis of CTTN gene amplification, comparing amplification within epithelial hyperplastic/dysplastic lesions and high-grade dysplasias/carcinoma in situ, to determine timing of amplification. Consistent with studies of 11q13 gene amplification being a later stage event [4], CTTN was only detected in the higher grade lesions [75]. Cortactin expression was also studied along with EGFR status in a series of HNSCC samples of different origins [76]. Cortactin overexpression correlated to higher TNM stage, histologic grade and was associated with decreased overall survival and increased local recurrence. However, patients that had both cortactin and EGFR overexpression did not have a different survival rate than those with cortactin only. This is surprising due to evidence that cortactin overexpression contributes to sustained EGFR surface expression by preventing ligand-mediated receptor downregulation [77] suggesting cortactin overexpression can be uncoupled from EGFR as reported in a subsequent study [78]. Cortactin overexpression in HNSCC does enhance c-Met surface expression, providing an additional mechanism for sustained c-Met signaling [77]. Another study focusing on laryngeal SCC found cortactin expression to be linked to both lymph node and distant metastases, and was identified as a predictor of poor prognosis in this HNSCC subtype [79]. The collective results from these recent studies strongly indicate that cortactin gene amplification and/or protein overexpression increases HNSCC aggressiveness. Although a late stage player in HNSCC progression, monitoring cortactin expression in HNSCC may also be useful in predicting invasive carcinoma and tumor recurrence.

### 3.2. CD44

Cell adhesion molecules (CAMs) are necessary for cell-cell or cell-extracellular matrix (ECM) contacts. The transmembrane glycoprotein CD44 is a CAM that binds hyaluronan (HA), a glycosaminoglycan component of the ECM and the primary ligand for CD44. The CD44 protein family consists of various isoforms that are the result of alternative splicing of exons 6-14 [80]. CD44 variants are often overexpressed in different cancer types, with overexpression correlating to poor patient outcome [80]. Standard CD44 (CD44s) and three variants, v3, v6 and v10, are overexpressed in HNSCC [81]. CD44s was overexpressed at a higher frequency in supraglottic laryngeal carcinomas of patients that were lymph node positive, although no significant differences were found between tumor stages and differentiation and CD44 expression [82]. The CD44 variants v3, v6 and v10 were identified in HNSCC samples from the oral cavity, oropharynx or larynx, with strong staining by IHC in both primary and lymph node metastases [81]. CD44 v3 and v6 were both associated with advanced T stage, while a strong v3 expression within primary tumors was related to lymph node metastasis and v10 expression related to distant metastases [81]. The same study also found CD44 mediated sensitivity to cisplatin *in vitro* and CD44 knockdown increased HNSCC cell death. Another interesting study analyzed blood for CD44 mRNA from patients with advanced HNSCC who had been treated with chemotherapy and radiotherapy to determine if there the presence of CD44 mRNA correlated with prognosis during the patient follow up period [83]. Quantitative RT-PCR detected mRNA in peripheral blood from patients and healthy volunteers, and elevated levels of CD44 mRNA in HNSCC patients correlated with the degree of lymph node involvement and recurrence. IHC analysis of tumor samples confirmed CD44 protein expression for all patients. These studies suggest a role for CD44 and specific splice variant isoforms in the regional and distant spread of HNSCC, with evidence for pre-treatment screening of CD44 being beneficial to determining prognosis and drug response.

### 3.3. Matrix Metalloproteinases

Invasion and metastasis of tumor cells requires proteolysis of the basement membrane and surrounding ECM. Matrix metalloproteinases (MMPs) provide cells with a mechanism to modulate the microenvironment through the breakdown of ECM molecules present in basement membranes and stroma [84]. Twenty three different MMPs are expressed in humans, with MMP-9 and MMP-14 (MT1-MMP) accepted as playing critical roles in HNSCC invasion and metastasis. MMP-9 is a secreted proteinase that utilizes CD44 as a docking site, allowing for its retention on the cell surface [84,85]. CD44s expression in supraglottic laryngeal carcinoma samples correlates with MMP-9 expression in lymph node positive patients [82]. Similar results were obtained in a second study of OSCC, linking MMP-9 expression to lymph node metastasis [86]. Expression of MMP-9 was also found to co-localize to the invasive front with CD44 of HNSCC patient samples, while normal mucosa showed little to no MMP-9 labeling [87]. A prospective study investigated whether serum MMP-9 levels of 161 patients with OSCC could be correlated to clinicopathological parameters [88]. Pre-treatment serum MMP-9 levels correlated to clinical stage and were also significantly higher in patients with lymph node metastasis, while a significant decrease in levels was seen after surgery. Presurgery levels of MMP-9 in patients who died during the study were found to be significantly higher than those who survived, linking serum MMP-9 to patient outcome [88]. The results of this study are comparable to an earlier study from 2005, which also investigated serum levels of MMP-9 in HNSCC patients before surgery [89]. Transmembrane MMPs are also important for the activation of secreted MMPs through the cleavage of secreted MMP proforms to generate a functional extracellular enzyme. Studies on supraglottic HNSCC patients demonstrate increased MT1-MMP expression compared to normal tissue. In this study, the level of MT1-MMP overexpression correlated to the depth of invasion, presence of lymph node metastasis and advanced clinical stage [90]. Patients with high MT1-MMP expression also had a poor prognosis. Interestingly, cortactin overexpression has been reported to enhance MMP-9 secretion and promote MT1-MMP surface expression in HNSCC cell lines, resulting in enhanced ECM degradation at plasma membrane structures known as invadopodia [91,92]. The pro-invasive function of combined MMP activity in HNSCC and other tumor types makes them useful as prognostic markers as well as attractive and important therapeutic targets. Despite previous failed attempts at targeted drug therapy [93,94], development of new generation MMP inhibitors is being pursued, where improved drug and trial design may yet result in the production of selective and effective anti-invasive drugs [94].

## 4. Tumor Microenvironment and Angiogenesis

HNSCC and other tumor types frequently exploit signals generated from cellular and non-cellular ECM components to promote tumor growth and dissemination. The process of new blood vessel formation (angiogenesis) is initiated in order to provide tumors with the means to supply nutrients to facilitate their growth, as well providing avenues for eventual metastasis through the vascular system.

### 4.1. Chemokines

Chemokines are small peptides that upon receptor binding act as chemoattractants, homing leukocytes to areas of inflammation [95,96]. Their roles in cell trafficking and angiogenesis help promote tumor growth, as evidenced by their overexpression in several different human cancers. The receptor CXCR4 and its ligand CXCL12 (also called SDF-1) is one chemokine pathway exploited by metastatic HNSCC [97]. A retrospective analysis by IHC of 30 patients with laryngeal and hypopharyngeal SCC showed a significant increase in CXCR4 expression in patients with positive lymph node and distant metastases compared to patients lacking metastatic disease [98]. This study also looked at CXCL12 expression and while higher in patients with metastasis, it was not statistically significant. Two other studies assessing the prognostic value of CXCR4 in OSCC also drew similar conclusions, finding a significant association of expression with lymph node metastasis [86,99]. One study examined expression of CXCR4 and another chemokine receptor, CCR7, which has been shown to activate the PI3K/Akt pathway in HNSCC, a pathway involved in cell growth, differentiation and survival [100]. Both chemokines were expressed at significantly higher levels in those patients with positive lymph node involvement compared with lymph node negative cases [100]. One notable difference was that CCR7 expression correlated to cases with advanced tumor stage, while CXCR4 was significantly higher in patients with distant metastases. A similar study also significantly associated CCR7 expression with lymph node metastasis, while CXCR4 expression was associated but not statistically significant [101]. CCR7 also positively associated with lymph node metastasis in patients with tonsillar SCC [102] and OSCC [103]. The influence of autocrine/paracrine activation of CCR7 was examined in another retrospective study of HNSCC and found higher mRNA expression of CCR7 and its ligands CCL19 and CCL21 in metastatic lymph nodes [104]. This study further concluded from additional *in vitro* and orthotopic mouse model studies that blockage of CCR7 impaired tumor cell proliferation and decreased resistance to cisplatin-induced apoptosis. The impact of chemokines and their receptors on HNSCC may be explained in part by their influence on MMPs [95,105]. CXCR4 increased HNSCC cellular invasion *in vitro* by upregulating expression of MMP-9 and MMP-13 [98,106]. High CXCR4 levels also correlate with high MMP-9 in OSCC patient samples [86]. CCR7 is typically involved in directing dendritic cells to peripheral lymph nodes [105]. The frequent expression of CCR7 in HNSCC and association with lymph node metastasis suggests that CCR7 expression provides tumor cells with a mechanism for direct lymph node infiltration [95,96,107,108]. These results suggest overlapping roles for chemokine receptors in HNSCC progression to metastatic disease, a consideration that may need to be taken into account for any future therapeutic targeting strategies.

### 4.2. VEGF/R

Vascular endothelial growth factor (VEGF) is a cytokine expressed by tumors that plays a key role in angiogenesis. VEGF performs its function through binding to one of the VEGF receptor family members, with VEGFR2 serving as the major receptor subtype in several different neoplasms [109]. The importance of VEGF in cancer progression has been well documented [110-114], and recent studies further demonstrate VEGF as a valuable prognostic marker for HNSCC. As a growth factor, circulating VEGF can be a useful marker for detecting advanced disease since circulating serum levels of VEGF in HNSCC patients before treatment was significantly higher when compared with non-cancerous individuals [115]. Patients with advanced T stage, lymph node metastasis and advanced disease stage also had significantly higher serum VEGF levels [115]. Another study involving the prognostic value of serum VEGF levels in nasopharyngeal carcinoma found a significant relationship between higher levels of VEGF and several clinicopathologic parameters, including T and N stage and distant metastasis [116]. Similar results were obtained in another prospective serum analysis of OSCC patients before and after treatment [88]. In a separate retrospective study, OSCC cases were analyzed for VEGF-C and VEGF-D expression by IHC [117], where increased staining intensity for these ligands significantly corresponded with lymph node involvement. In addition, lymphatic vessel density (LVD) was also evaluated in this report, where high LVD correlated with VEGF-C/VEGF-D expression. Another study evaluated the relationship between lymph node metastasis and VEGF. Positive IHC expression of VEGF and Notch1, a receptor capable of promoting transcription of genes involved in cellular proliferation [118] was observed in patients with early SCC of the tongue. High Notch1 expression was also more frequent in patients with lymph node involvement. Another analyzed variable in this study was the distance of tumor cell invasion from the surface mucosa. A greater invasion depth was found in patients with elevated VEGF expression compared with cases containing normal VEGF levels [110]. In a separate study focusing on the role of angiogenesis in early SCC of the tongue, VEGF expression was found in 74% of analyzed patient samples, where it correlated with increased tumor size, disease stage, lymph node invasion, tumor recurrence and distant metastases [119]. Retrospective mRNA analysis of VEGF-C and VEGFR3 in locoregionally relapsed HNSCC revealed a significant association of high mRNA levels and relapse beyond the primary tumor [120]. While the majority of studies on VEGF expression supports a pro-metastatic role, there have been some reports where such a correlation between VEGF expression and advanced tumor stage is not evident [121,122]. However, only laryngeal SCC was analyzed in one of these studies, with the data suggesting patients with advanced disease and lower VEGF expression would benefit the most from induction chemotherapy [121]. In a separate report, VEGF expression did not predict metastasis in early (T1 or 2) stage OSCC [122]. Collectively these studies suggest VEGF and/or VEGFR status is useful as a pro-metastatic marker in HNSCC, and may also serve to predict relapse and treatment response.

## 5. Transcription Factors

Transcription factors are required for normal cellular homeostasis. Dysregulation of transcription factor expression is a major contributor in initiating cancer and driving tumor progression. Several transcription factors have been found to regulate expression of target genes involved in promoting HNSCC lymph node metastasis. The pleiotropic effect of these transcriptional regulators on diverse signaling pathways makes them rational targets for therapeutic intervention in HNSCC and other cancer types.

### 5.1. NF-κB

Nuclear factor-kappa B (NF-κB) is part of a family of transcription factors that regulate genes needed for most aspects of neoplastic transformation [123,124]. Inflammation has been linked to cancer progression [125], and as a proinflammatory transcription factor NF-κB is often found expressed in most tumor types [124]. Specific to HNSCC, analysis of tumors from varying primary sites as well as matched lymph node metastases showed positive nuclear NF-κB expression, and was found with a greater significant frequency in primary tumors with metastasis [126]. NF-κB expression levels were highest in the nodal metastases evaluated in this study. A study of laryngeal cancer patients also reported a connection between NF-κB expression and lymph node metastasis, as well as T stage and overall survival [127]. A retrospective study of early-stage laryngeal cancer correlated NF-κB expression to local recurrence in patients resistant to radiotherapy [128]. In addition, patients with recurrence and positive NF-κB expression in pretreatment tumors showed enhanced expression in recurrent tumors, while those with recurrence but without expression before treatment became NF-κB-positive. NF-κB has been shown to regulate other proteins involved in HNSCC cellular proliferation and metastasis, such as survivin [55], Twist1 [129], Snail [130], VEGF and MMP-9 [126], as well as other targets in HSNCC [123,124]. It has also been demonstrated that NF-κB expression can be regulated by chemokines or interact with other transcriptional regulators (Hif-1α) [131-133], making NF-κB a central player in the development and spread of HNSCC. Therapeutic targeting of NF-κB would therefore potentially disrupt multiple pathways important in HSNCC.

### 5.2. Regulators of EMT

Epithelial-mesenchymal transition (EMT) is a process in which cells lose epithelial traits and obtain a mesenchymal phenotype. While a normal part of embryonic development, EMT is a major mechanism that drives cancer development and progression. Several proteins involved in the induction of EMT in cancer have been identified [134,135]. Among these are the transcription factors Twist and Snail. Twist belongs to the basic helix-loop-helix family of transcriptional regulatory proteins [136]. Upregulation of Twist expression has been shown to promote EMT in breast cancer, while downregulation suppresses metastasis [137]. In HNSCC, Twist has been correlated to lymph node metastasis through tissue microarray screening [101]. Higher Twist expression was also observed in metastatic samples when compared to primary tumors, significantly correlating with reduced survival [138]. Another study correlated high tumor grade to Twist1 expression in HNSCC cases and while not statistically significant, Twist1 expression was associated with poor prognosis [139]. Several *in vitro* studies have implicated Twist expression in the acquisition of chemotherapeutic resistance for various cancer types [90,129,140]. One of these was nasopharyngeal carcinoma, where decreased Twist expression by RNAi enhances sensitivity to chemotherapeutic compounds such as taxol and cisplatin [90,129,140].

The transcription factor Snail is a zinc-finger transcriptional repressor that induces EMT by suppressing expression of E-cadherin, a component of adherens junctions that maintains epithelial cell-cell adhesion [136]. Expression of Snail was also found at higher levels in metastatic HNSCC samples [138] and was positively associated with higher-grade tumors, lower survival rates [141,142], increased invasion depth and development of metastases [142]. Additionally, another earlier retrospective study found higher Snail expression correlated with cervical lymph node and distant metastasis. When co-expressed with NBS1, Snail expression resulted in higher probability of metastasis and shorter survival periods [45]. Like Twist, Snail has also been associated with chemoresistance [143]. Snail promoted cisplatin resistance in HNSCC cell lines via upregulation of excision repair cross-complementation group 1 (ERCC1), a protein important in nucleotide excision. IHC analysis of HNSCC patients who had undergone cisplatin treatment revealed a higher risk of resistance with Snail expression. Twist1 was also evaluated in this study and was correlated with greater resistance [143]. Given their function in HNSCC and other tumor types, Twist1 and Snail expression levels are likely good candidates for monitoring the invasive and metastatic potential of primary HNSCC, and may be useful in predicting patient response to chemotherapy.

### 5.3. Hif-1α

In response to the low oxygen (hypoxic) environment present in primary tumors as they proliferate and increase in size, tumor cells activate hypoxia-inducible factor 1 (HIF-1) to upregulate proteins necessary for preventing cell death [144,145]. HIF-1 is a heterodimeric protein consisting of an alpha and a beta subunit [144]. The HIF-1 alpha subunit is needed for HIF-1 to function as a transcription factor, and responds to cellular oxygen levels by activating transcription of genes such as VEGF, platelet-derived growth factor (PDGF) and transforming growth factor-α (TGF-α) to survive under hypoxic conditions [144]. In OSCC, high HIF-1 alpha expression was correlated to worse outcome in metastatic OSCC samples [138,146]. Overexpression of HIF-1 alpha was frequently observed in HNSCC patients with lymph node metastasis, and was significantly higher when compared with node-negative cases [146]. Hypoxic tumor cells are known to influence other factors required for HNSCC cell survival and growth including EMT, ECM invasion and angiogenesis [147]. HIF-1 alpha performs these functions through regulation of expression and/or activity of multiple proteins, including Twist, MMP-2, MMP-9, VEGF and CXCR4/SDF-1 [147,148]. Hypoxia and HIF-1 alpha have been cited as one of the major causes of drug resistance to anti-angiogenic therapies in many human cancers [149]. These studies suggest that HIF-1 alpha expression may be a potential candidate to serve as a pro-metastatic biomarker in HNSCC and also predict treatment response.

## 6. HPV and HNSCC

Increased risk of HNSCC has largely been attributed to tobacco exposure and alcohol use; however in recent years, studies have also implicated human papillomavirus (HPV) infection as an additional risk [4,150,151]. Approximately 20-25% of HNSCC are HPV-positive, with the majority of these cases arising in the oropharynx [4,150,151]. The HPV proteins E6 and E7 are key players in carcinogenesis, causing the destabilization and degradation of cell cycle regulators p53 and pRb [150,151]. As a result, deregulation of cell cycle checkpoints and downregulation of other cell cycle regulatory proteins occurs, leading to genomic instability and uncontrolled proliferation [150,151]. There are multiple differences between HPV-positive and HPV-negative HNSCC, making them clinically distinct and requiring different management strategies. In HPV-positive HNSCC cases, genome-wide alterations in DNA copy number that are frequent in HPV-negative tumors are fewer in number; there are also fewer TP53 mutations, and epigenetic changes such as p16^INK4A^ gene silencing due to downstream Rb degradation [4,151]. Another major difference is in cell cycle regulatory pathways, with upregulation of cyclins D and E being a common occurrence in HPV-positive tumors. An important distinction to note is the expression of p21^WAF1/Cip1^, a cell cycle regulator that normally functions to promote cell cycle arrest through binding and interfering with cyclin-dependent kinases (CDKs) 1 and 2, as well as proliferating cell nuclear antigen (PCNA) [152] to produce cell senescence. p21^WAF1/Cip1^ also promotes resistance to apoptosis [153] and is either up or down regulated depending on cancer type, giving it dual oncogenic and tumor suppressor properties [152]. In a retrospective study involving 117 patient samples, p21^WAF1/Cip1^ was overexpressed in HNSCC samples from pharynx and larynx, and its increased expression was correlated with lymph node metastasis, locoregional relapse and decreased survival rate [154]. HPV status was not determined in this report. In a more definitive study of HPV-positive HNSCC, p21^WAF1/Cip1^ overexpression was associated with favorable outcome [155]. In general, HPV-positive cancers have better prognosis and response to radio- and chemotherapies, with fewer distant metastases when there has been no tobacco exposure [4,151,156]. For these reasons, HPV-positive HNSCC should be treated as a separate subtype with specific biomarkers and warrants determination of HPV as standard practice in order to accurately format appropriate treatment strategies.

## 7. Conclusions

Development of lymph node metastases remains a major prognostic factor in HNSCC. Many patients present with clinically advanced disease, where surgery, radiation and chemotherapy are the standard of care. Given the highly disfigurative nature of HNSCC surgical treatment and typically repeated exposure to high-dose radiation, identification of primary HNSCC tumors with enhanced metastatic potential by molecular means can aid clinicians in tailoring appropriate treatment strategies, especially in cases that have no apparent nodal involvement. Current histological procedures can be limited in their ability to detect nodal metastasis, highlighting a need for detailed, accurate molecular analysis of individual HNSCC tumors to determine specific deleterious protein expression patterns. Such a molecular analysis would theoretically result in an increased ability to identify patients with a greater risk for metastasis formation, allowing for rational treatment design to be tailored for the best possible patient outcome [157].

Early detection of oral premalignant lesions is one route to improving patient prognosis. Markers predicting progression of these lesions to cancer have not been extensively studied, however a recent gene expression profiling study revealed a signature useful in predicting OSCC development [158]. The biomarkers of focus here have been more extensively characterized in tumor spread beyond the primary site, however it would be interesting to find out if they could also be useful in identifying precancerous lesions at risk for progression to carcinoma. Some of these proteins are more useful in later stages of HNSCC. Expression of cortactin, CD44, NBS1, CXCR4, Snail and VEGF in patients with metastatic disease has been correlated to the development of distant metastases. These patients may benefit from induction and maintenance therapy in order to prevent spread below the clavicles. Monitoring serum levels of HGF, MMPs and VEGF has also been shown to be beneficial in predicting patient outcome, and further studies involving prospective analyses could provide an easier route to identification of patients at higher risk for metastasis.

**Figure 1.**
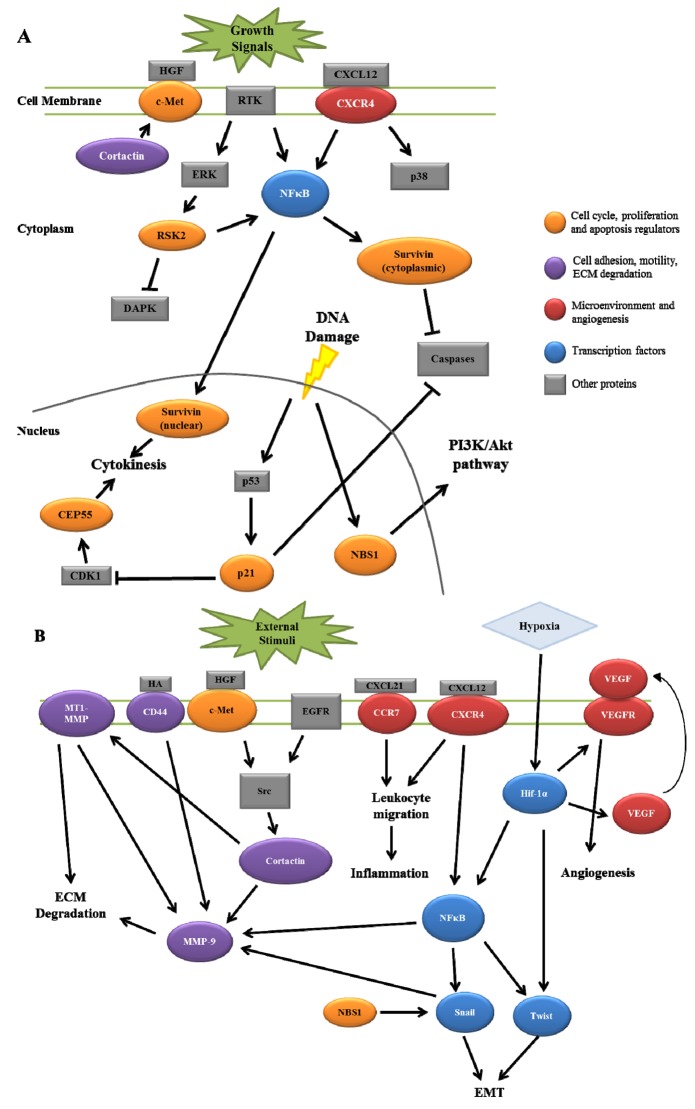
Potential interactions between recently identified biomarkers involved in HNSCC lymph node metastasis. (A) Putative interactions between proteins involved in cell cycle regulation, cell proliferation and apoptosis. (B) Potential interactions highlighted for mediators of cell motility, adhesion, ECM degradation and tumor microenvironment.

HNSCC invasion and nodal metastasis is a complex process involving multiple signaling pathways and protein components. As reviewed here, several proteins have recently been characterized that give insight into HNSCC progression that have been documented to interact in signaling pathways ultimately resulting in lymph node metastasis. Figure 1 summarizes the potential interactions between these signaling pathways. Combinations of multiple protein expression patterns may potentially produce an accurate lymph node metastasis signature that could serve as a predictive tool for analyzing patient tumors. While the implementation of such a signature would require further validation in both experimental and clinical settings, the outcome of such work would provide an improved understanding of HNSCC as a disease, with the combined goal of enhancing overall patient quality of life.

## Figures and Tables

**Table 1.** Recently identified biomarkers involved in lymph node metastasis.

**Biomarker**	**Cellular Function**	**Relevant References**
c-Met	Proliferation	[23]
CEP55	Cell cycle regulation, cytokinesis	[39,40]
NBS1	Cell cycle regulation, DNA double-strand break repair	[43,45]
Survivin	Inhibitor of apoptosis	[51,55,61]
RSK2	Cell cycle regulation, proliferation, apoptosis	[65]
Cortactin	Cell motility and invasion	[74-76,79]
CD44	Cell-cell and cell-matrix adhesions	[81-83]
MMP-9	ECM degradation	[81,88]
MT1-MMP	ECM degradation	[90]
CXCR4	SDF-1 chemokine receptor, chemoattraction	[98-101]
CCR7	Chemokine receptor, chemoattraction	[100,101,103]
VEGF/R	Angiogenesis	[110,115,117,119]
NFκB	Proinflammatory TF	[126,127]
Twist	TF, regulator of EMT	[101,138]
Snail	TF, regulator of EMT	[138,141,142]
Hif-1α	TF, hypoxia	[138,146]
p21^WAF1/Cip1^	Cell cycle regulation, proliferation, apoptosis	[154,155]

MMP: matrix metalloproteinase; ECM: extracellular matrix; TF: transcription factor;

EMT: epithelial-mesenchymal transition.
